# Impingement of the ankle joint—a systematic review on the expected outcome

**DOI:** 10.1186/s12891-025-08785-8

**Published:** 2025-07-12

**Authors:** Maximilian A. Hamberger, Maximilian M. Saller, Wolfgang Böcker, Hans Polzer, Sebastian F. Baumbach

**Affiliations:** 1 Department of Orthopaedics and Trauma Surgery, Musculoskeletal University Center Munich (MUM), University Hospital, LMU Munich, Ziemssenstraße 5, Munich, 80336 Germany; 2 Department of Orthopaedics and Trauma Surgery, Musculoskeletal University Center Munich (MUM), University Hospital, Ludwig-Maximilians-University (LMU), Fraunhoferstraße 20, Planegg/Martinsried, 82152 Germany; 3 OrthoPlus Munich, Lenbachplatz 2a, Munich, 80333 Germany

**Keywords:** Ankle impingement, Impingement syndrome, os trigonum, Return to sports, Stieda process

## Abstract

**Background:**

Impingement syndrome of the ankle is a multifaceted condition. It can affect all regions within the ankle joint and can be caused either by bony prominences or soft tissue. Exact numbers on the incidences are missing, prevalence rates up to 60% are estimated in certain risk groups. Previous systematic reviews have focused on specific anterior or posterior ankle impingements. Still, a comprehensive overview over impingement syndromes of the ankle and the outcome to be expected is missing. This systematic review aims at giving a comprehensive overview on studies reporting on the outcome of any kind of ankle impingement.

**Methods:**

A systematic review was conducted, based on a thorough search strategy in four databases following the PRISMA guidelines. Included were all studies reporting on the outcome for any sort of ankle impingement. Assessed data were the level of evidence, study details, general demographics, injury details, surgical treatment, postoperative treatment protocol, follow-up time, complications, and outcome. Analyzed were comparable outcome parameters presented in at least three studies.

**Results:**

Out of 3083 screened abstracts, 85 studies were eligible for qualitative and 37 studies for quantitative analysis. The AOFAS was assessed by 21 studies, with significant postoperative improvement in all studies. In all but two studies, mean postoperative AOFAS scores above 80 points were achieved. The VAS pain on a 10-point Likert scale was reported in 12 studies and improved in all. Return to sports was reported as a percentage/duration in 17/14 studies. At least 70% of patients returned to sports in all but one study.

**Conclusions:**

This study is the first to provide a comprehensive overview over the expected outcomes following surgical treatment of any ankle impingement syndrome. The great heterogeneity of the studies did not allow a pathology-specific comparison. Surgery apparently improves the symptoms considerably, but the outcome varies between the different types of impingements, with a posterior os trigonum impingement resulting in the most favorable outcomes.

**Trial registration:**

The study was a-priori registered at Prospero (#CRD42022354685) on August 19th, 2022.

**Level of evidence:**

Level 1 Systematic Review.

**Supplementary Information:**

The online version contains supplementary material available at 10.1186/s12891-025-08785-8.

## Background

Tibiotalar impingement is characterized by the entrapment of soft tissue or bone at the ankle joint, leading to pain and/or reduced range of motion [1, 2]. Symptoms typically occur in terminal motion, present with a gradual onset, and have been associated to repetitive stress/microtrauma [3]. Ankle impingement syndromes are most often classified according to their location (anterior / posterior; medial / central / lateral) and the interposing structure (soft tissue / bony, os trigonum, Stieda process). Exact numbers on the incidences are missing, but various studies suggest a considerable higher incidence in professional athletes [4, 5]. The two most common impingement syndromes are the anterior bony ankle impingement and the posterior bony os trigonum impingement. Anterior bony impingement occurs in up to 60% of professional soccer players [4] and has therefore received the eponym soccer’s ankle [6]. Posterior ankle impingement caused by an os trigonum has been reported in a high percentage of female ballet dancers, fast bowlers, and soccer players [5, 7].

The diagnosis of any impingement syndrome is predominantly based on the clinical examination, which should be verified by imaging modalities [5]. Initial treatment comprises of physiotherapy, anti-inflammatory drugs, taping, and possibly corticosteroid injections [8–10]. In case of failed non-operative treatment, open, arthroscopic, or endoscopic surgical intervention should be considered [10]. Previous systematic reviews have focused on specific anterior [11, 12] or posterior [9, 13] ankle impingements. Up to now, a comprehensive systematic review including all types of ankle impingements is missing.

The aim of this study was to provide a systematic overview on all studies reporting on the outcome of any kind of ankle impingement.

## Methods

The systematic review was conducted according to the PRISMA guideline [14]. The study was a-priori registered at Prospero (#CRD42022354685).

### Search strategy

MEDLINE (PubMed), Scopus, Central and EMBASE were searched from inception to August 19^th^, 2022. The review question was framed according to the PICOS criteria (Table 1). The search strategy was built upon the principal strategies of Impingement AND Ankle Joint. The entire search strategy is presented in Supp. 1. A grey literature search for conference proceedings was performed in Scopus and EMBASE and a general search in OpenGrey. In addition, all references of the studies included, and systematic reviews identified by the systematic search were hand searched to identify studies missed by the initial search.

**Table 1:** PICOS criteria defining the inclusion and exclusion criteria

Population	Adult patients suffering from any sort of ankle impingement, including posttraumatic arthrofibrosis.
Intervention	Any sort of treatment, i.e. surgical or conservative, open or arthroscopically/endoscopically.
Comparison	Not applicable
Outcomes	Any objective outcome measure, including ROM and PROMs.
Study	Any study, independent of its design, but no case reports (<6 patients).

### Study selection

Each database was searched separately, and the resulting datasets were exported to one Endnote™ file (Vs. 20.1, Fa. Clarivate). Following the removal of duplicates, the final dataset was exported to Covidence™ (Melbourne, Australia). The screening was done in duplicate by two independent reviewers for both title and abstract as well as full-text screening stages. In case of disagreement, the publication was forwarded into the full-text screening stage. At that stage, disagreements were resolved by discussion between both reviewers and a third blinded reviewer.

### Data extraction

Data extraction was performed by two reviewers and based on standardized data extraction sheets in Microsoft® Excel® (Vs. 16, Microsoft, Redmond, Washington, USA). The data extracted were level of evidence, study details, general demographics (age, sex, etc.), injury details (acute < 6 weeks vs. subacute ≥ 6 weeks to 3 months vs. chronic ≥ 6 months; classification and distribution of injuries), surgical treatment (closed, open, arthroscopically/endoscopically), follow-up time, and outcome. The outcome was defined as the patient rated outcome, assessed by patient rated outcome measures or visual analogue scale.

### Risk of bias assessment

A risk of bias assessment was performed for all studies eligible for a cumulative analysis. The quality assessment was conducted by two of the authors independently. The level of evidence was assessed as recommended by Wright et al. [15]. The risk of bias was assessed using the Methodological Index for Non-Randomized Studies (MINORS), which can be applied to randomized and non-randomized studies [16]. The risk of bias assessment for each study is presented in Supp. 3.

### Data analysis

All studies eligible were first be grouped per the type of impingement. These were subdivided into anterior / posterior and soft tissue / bony impingement. The subgrouping was conducted as specific as possible. Paper presenting combined results on various pathologies were excluded as they did not allow for a specific analysis. Next, all reported outcome measures were extracted. In case three studies facilitated the same outcome scores, they were eligible for a cumulative analysis. If a sufficient degree of homogeneity within the study was observed, a meta-analysis was conducted, otherwise the results were presented cumulative, without calculating an overall effect. Cumulative data presentation in Fig. 3 was conducted using RStudio (version 4.3.0, Boston, MA, USA).

## Results

**Fig. 1:**
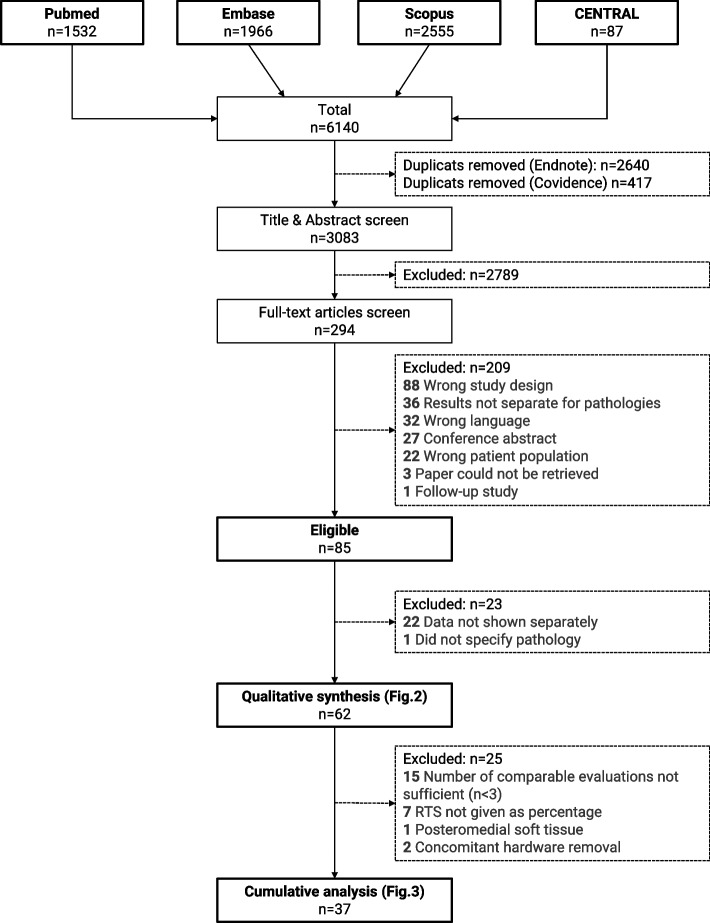
Flow-chart illustrating the study selection process

The study selection process is outlined in Fig. 1. In brief, out of 3083 screened abstracts, 85 studies were eligible for further analysis. 22 studies did not present their outcome data separately for the individual pathologies and were therefore excluded. One further study did not specify the impingement causing pathology and was therefore also excluded. The remaining 62 studies were included in the quantitative analysis.

**Fig. 2:**
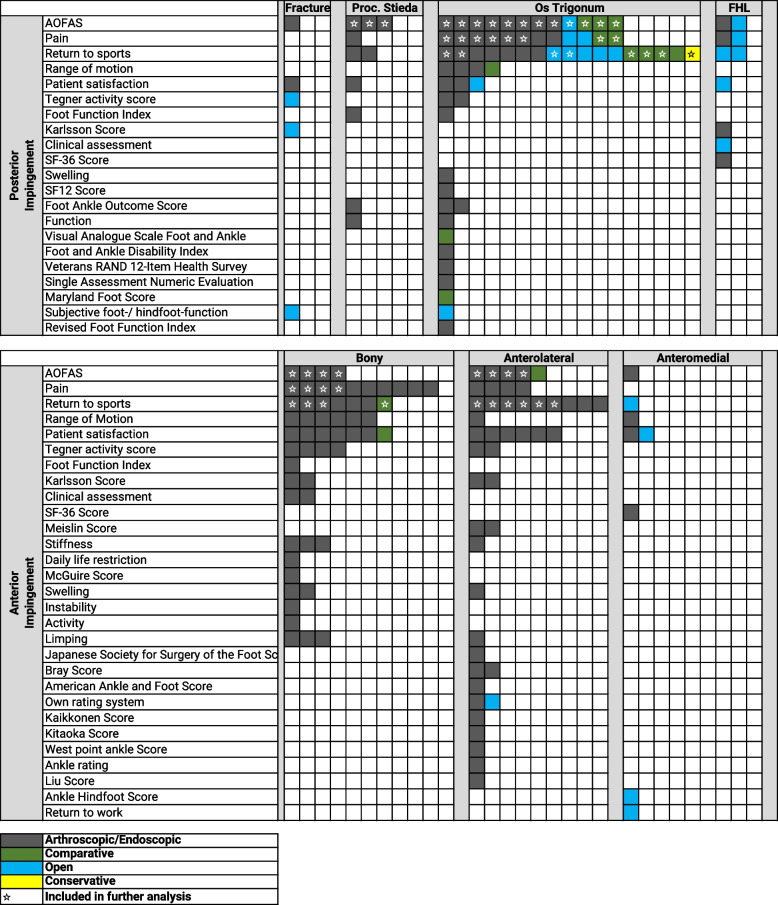
Illustration of the outcome parameters assessed per the specific pathologies in studies reporting the outcomes for each pathology separately. Legend: Abbreviations: Proc. = Processus, FHL = Flexor hallucis longus, AOFAS = American Orthopaedic Foot and Ankle Society, SF = Short Form Survey. Pain: Studies used various different scales to report pain levels. Studies using a 10-item Likert scale were eligible for a cumulative analysis and these were marked with a star. ☆: Marks all studies with identical outcome parameters, which were reported in at least three studies for an individual pathology, that were therefore further analyzed in the cumulative/quantitative analysis

Then, these 62 studies, including the assessed outcome parameters, were grouped per the location (posterior or anterior) and the type of impingement (posterior: fracture, proc. Stieda, os trigonum, FHL; anterior: bony, soft tissue anterolateral or anteromedial). Anterior bony impingement was not further subdivided into tibial, talar, or combined osteophytes, as only one study reported the outcomes individually [17]. The final, grouped list is presented in Fig. 2. Overall, the studies showed a great heterogeneity which prohibited a further meta-analysis.

To present an overview for the expected outcome per the individual pathologies, the authors identified all studies, in which the same outcome parameter for the same pathology was assessed in at least three studies (Fig. 2; stars). These outcome parameters were the AOFAS, the VAS on a 10-point Likert scale, and return to sports. Overall, 37 studies were eligible for this cumulative qualitative interpretation (Fig. 1).

**Fig. 3:**
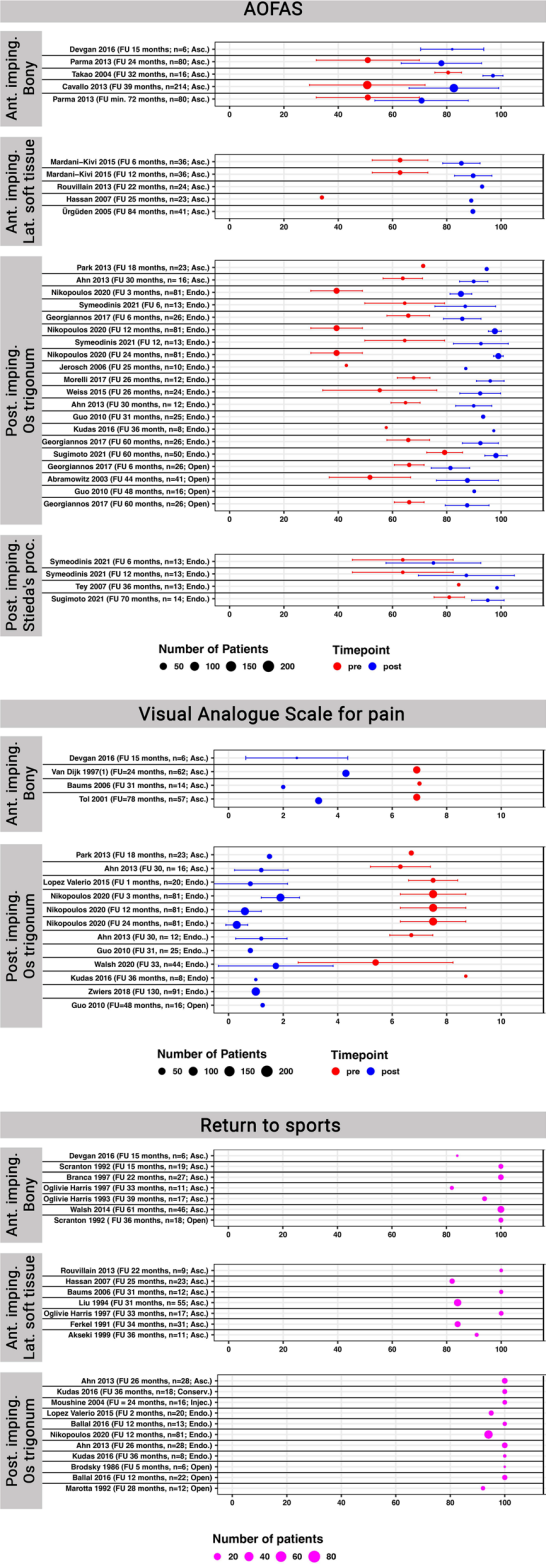
Bar plots illustrating the outcome per the different pathologies and treatment modalities in order of the reported follow up. If studies reported multiple follow-ups, all of them were included. Legend: Abbreviations: FU = follow-up, *n* = number of patients, Ant. = Anterior, Post. = Posterior, Lat. = Lateral, imping. = impingement, Asc. = Arthroscopic, Endo. = Endoscopic, Open = Open surgery, Conserv. = Conservative, Injec. = Injections

In Fig. 3, the outcome parameters (AOFAS, VAS, Return to sports) are shown separately per the pathology addressed, the surgical technique, and the follow-up period. If studies reported multiple follow-ups, all follow-ups were included. The demographic details and complications are listed in Supplement 2. The patients were on average 28 ± 7 years old. 30% were female. Overall complications for anterior arthroscopy were 5%, for anterior open procedures 0%, for posterior arthroscopic procedures 3%, for posterior endoscopic procedures 5%, and for open posterior procedures 15%. The majority of the complications following open posterior procedures were (temporary) sural nerve affections. Due to varying definitions, the complications could not be differentiated between major or minor complication.

The AOFAS score was assessed in 21 studies (23 study-arms) [10, 18–37]. Four studies each reported on anterior bony- or lateral soft tissue impingement, all facilitating arthroscopy. Twelve studies reported on posterior os trigonum impingement, facilitating arthroscopic, endoscopic and open procedures. Finally, three studies reported on posterior impingement due to a Stieda’s process, all facilitating an endoscopic approach. As illustrated in Fig. 3, the outcome improved in all studies between pre- and postoperative. For anterior soft tissue impingement and os trigonum the final outcome scores were above 80 points on average in all studies. Slightly worse outcomes were found for anterior bony impingement and posterior Stieda’s process impingement.

The VAS score for pain on a 10-point Likert scale was reported in 12 studies (12 study-arms) [18, 26–28, 33, 34, 38–43]. Four studies, all facilitating an arthroscopic procedure, reported on the outcome of anterior bony impingement. Eight studies reported on the outcome of a posterior impingement caused by an os trigonum, facilitating arthroscopic, endoscopic, or open procedures. Again, all comparative studies showed an improvement between pre- and postoperative. Similar to the AOFAS scores, anterior bony impingement resulted on average in worse outcome scores compared to an os trigonum.

Return to sports was reported as percentage in 17 studies (22 study-arms) [23, 24, 27, 28, 34, 38, 41, 44–53]. Five studies facilitating arthroscopic and open procedures reported on anterior bony impingement. Seven studies, all facilitating an arthroscopic procedure reported on anterior, lateral soft tissue impingement. Eight studies reported on a posterior bony impingement due to an os trigonum, facilitating conservative, arthroscopic, endoscopic, or open procedures. The return to sports rate was more than 80% in all studies. Still, the resection of a symptomatic os trigonum again seems to result in slightly favorable outcomes compared to an anterior bony or soft tissue impingement.

Fourteen studies (19 study-arms) [18, 23, 26–28, 32–34, 36, 38, 44, 50–52] reported on the duration until full return to sports. The reported period varied between a minimum mean of 36.3 days (24–42 days) [34] after conservative therapy of an os trigonum and a maximum mean of 4.66 months (SD 2.5 months) [18] after arthroscopic surgery of anterior bony impingement. On average the mean time to return to sports was 10.5 weeks [18, 23, 26–28, 32–34, 36, 38, 44, 50–52].

## Discussion

The herein conducted systematic review confirms that surgical treatment leads to symptom relief in patients with ankle impingement syndromes. While the overall evidence, based on 37 quantitatively analyzed studies, supports consistent improvements in function and pain, the magnitude of benefit appears to vary depending on the type of impingement. Patients with a posterior os trigonum impingement experienced the most favorable outcomes. This indicates a possible pathology-specific gradient in surgical success. These findings underline the overall effectiveness of surgery while highlighting the importance of different treatment expectations depending on the underlying type of impingement.

The AOFAS overall showed a considerable improvement per the preoperative and postoperative values, independent of the individual pathology (Fig. 3). In all but two studies [19, 29], the reported mean postoperative values were above 80 points. Contrary, the preoperative AOFAS scores varied considerably between 34 [24] and 84 points [37]. The AOFAS improvement ranged therefore between 14 [37] and 46 points [28]. Anterior bony ankle impingement overall tended to result in inferior outcomes, compared to the other pathologies analyzed. Os trigonum impingement was the only pathology for which different surgical procedures were applied, i.e. arthroscopy (*n* = 2) [26, 27], endoscopy (*n* = 10) [10, 27–35], or open (*n* = 3) [10, 33, 36]. Still, the postoperative AOFAS scores were comparable. A recent prospective cohort study on 6754 professional soccer players also reported on superior outcomes following treatment of posterior- compared to anterior ankle impingement. [4] Another study comparing anterior soft tissue impingement to anterior bony impingement could not show any significant differences between the two entities regarding AOFAS, VAS pain score, return to sports time and range of motion [18]. Possible reasons for the inferior AOFAS scores for anterior bony impingement could be their pathogenesis or accompanying intraarticular pathologies (e.g., chondral defects). Anterior bony impingement is believed to either result from repetitive stresses to the joint capsule [6, 17, 47, 54] or a (multidimensional) ligamentous instability of the ankle joint [55, 56]. Accompanying chondral defects have been correlated to an inferior outcome [23, 57]. Out of the four studies included in the current analysis [18–21], three reported on accompanying chondral lesions [18, 19, 21]. Solely addressing the anterior bony impingement therefore might not solve the often multidimensional problems in those joints.

Two other systematic reviews have assessed the postoperative AOFAS scores after anterior [12] and posterior [9] ankle impingement. The review on anterior ankle impingement [12] included 7 studies with postoperative AOFAS scores ranging from 83.5 to 92 points. Out of those 7 studies, one study group reported on bony, six on soft tissue impingement. The systematic review on posterior ankle impingement [9] included 32 studies and reported superior outcomes for endoscopic (AOFAS: 94 points) compared to open (AOFAS: 88 points) treatment. Out of the 32 studies, 14 reported on the resection of an os trigonum, 15 on bony impingement and seven on soft tissue impingement. These two reviews highlight a frequently encountered problem in systematic reviews and meta-analysis. Authors are often too liberal on which studies are included and the way they are analyzed.

Pain was assessed per a 10-item Likert visual analogue scale (VAS) in four studies reporting on anterior bony impingement [18, 40–42] and in eight studies for an os trigonum [26–28, 33, 34, 38, 39, 43]. Overall, preoperative pain scores ranged between 6 and 8 in all but two studies [34, 43]. Like the AOFAS scores, surgical treatment of anterior bony impingement tended to result in inferior VAS scores (2–4 points), compared to an os trigonum resection (0–2 points). Previous systematic reviews have not focused on the VAS pain scale, therefore comparable data is missing.

All patients returned to previous levels of activity in one [44] out of four studies [44–47] for anterior bony impingement and in no one of six studies [23, 24, 41, 46, 48, 49] for anterior soft tissue impingement. For an os trigonum, in eight studies with overall eleven study groups [27, 28, 34, 38, 50–53], nine study groups of six studies [27, 34, 50–53] had return to previous level of activity rates of 100%. This observation is comparable with the above-mentioned cohort study on professional soccer players [4]. Another systematic review comparing open to endoscopic removal of posterior ankle impingement described no significant difference regarding return to sport rates related to kind of treatment [9].

For Anterior soft tissue impingement and Posterior Stieda process impingement only a single surgical approach was used in the studies analyzed, i.e. arthroscopy or endoscopy. For Anterior bony impingement all but one study [44] used only arthroscopy. Surgical techniques for the os trigonum were arthroscopy (*n* = 2) [26, 27], endoscopy (*n* = 10) [10, 27–35], or open (*n* = 3) [10, 33, 36]. The herein conducted qualitative analysis revealed no obvious differences per the AOFAS, pain, or return to sports. Zwiers et al. [9] conducted a meta-analysis comparing open to endoscopic surgical treatment of posterior ankle impingement. They also found no significant differences for the AOFAS, time to return to activity, and complication rates.

The most pronounced limitation of the herein conducted systematic review was the encountered study heterogeneity. Although 85 studies were eligible for qualitative analysis, more than 50% of the initially identified studies had to be excluded as they did not differentiate between the different pathologies. The remaining 37 studies eligible for quantitative analysis still varied per the type of outcome parameters assessed, the treatment applied, and the individual follow-up periods. Out of 40 different outcome parameters reported in these studies, only three were reported in at least three studies, i.e. the AOFAS (21 studies), the VAS score for pain on a 10-point Likert scale (12 studies), and the percentage of patients returning to sports (17 studies). But even for those parameters, the data quality was insufficient to conduct a meta-analysis. A possible bias the authors did not control for is the differentiation between regular patients and professional athletes. These populations vary considerably per their baseline physical conditions, the available rehabilitation structures, and their desired level of activity [10, 31, 34, 38, 44]. Finally, the current systematic review did not evaluate the individual complication rates. Although they likely affect the outcome, the individual definition of what a complication was varied considerably between the studies included. Therefore, the authors decided not to further analyze these.

Despite the above outlined limitations, several strengths of the current study must be mentioned. The systematic review was conducted per the current guidelines and a formally built, comprehensive search algorithm was used. It is the only systematic review investing all types of ankle impingement and clearly differentiating the different pathologies of anterior and posterior ankle impingement. Therefore, it does provide a comprehensive overview over the expected outcome of all different types of ankle impingement.

Still the herein presented, cumulative data interpretation does highlight a devastating heterogeneity in the studies available. We as a research community must strive for a higher standardization of the research performed. This includes the parameters assessed, the scores used, the time points of assessment and the data format.

## Conclusion

This study is the first to provide a comprehensive overview over the expected outcomes following surgical treatment of any ankle impingement syndrome. The great heterogeneity of the studies did not allow a pathology-specific comparison. Surgery apparently improves the symptoms considerably, but the outcome varies between the different types of impingements, with a posterior os trigonum impingement resulting in the most favorable outcomes.

## Supplementary Information

Supplementary Material 1. Search strategy.

Supplementary Material 2. Study list with demographic details and complications.

Supplementary Material 3. Risk of bias assessment (MINORS).

Supplementary Material 4. PRISMA Checklist for Systematic Reviews.

## Data Availability

All data used for this systematic review is available on MEDLINE (PubMed), Scopus, Central and EMBASE. All search terms used are available in Supplement 1. All data generated or analyzed during this study are included in this published article, figures and supplementary files. The overall raw-table commenced from the single data sheets can be provided on request.
