# Supplementary material for: Impingement of the ankle joint—a systematic review on the expected outcome
Source: BMC Musculoskelet Disord. 2025 Jul 12;26:678. doi: 10.1186/s12891-025-08785-8 (PMC12254996; doi:10.1186/s12891-025-08785-8)
Supplement: Supplementary file 1 — Supplementary Material 1. Search strategy. [file 12891_2025_8785_MOESM1_ESM.docx]

**Supplement 1**

**Impingement of the Ankle Joint - A Systematic Review on the Expected Outcome.**

Maximilian A. Hamberger, Maximilian M. Saller, Wolfgang Böcker, Hans Polzer, Sebastian F. Baumbach

**Search strategy**

**PubMed 🡪 1532 hits**

(Impingement[Title/Abstract] OR Imping*[Title/Abstract] OR „Os trigonum"[Title/Abstract] OR „entrapment"[Title/Abstract] OR „entrap*"[Title/Abstract] OR Arthrofibrosis[Title/Abstract] OR scar[Title/Abstract]) AND ("Ankle Joint"[Mesh] OR "Ankle"[Mesh] OR "Ankle joint"[Title/Abstract] OR ankle[Title/Abstract])

**Scopus 🡪 2555 hits**

TITLE-ABS-KEY(Impingement OR Imping* OR „Os trigonum" OR „entrapment" OR „entrap*" OR Arthrofibrosis OR scar) AND TITLE-ABS-KEY("Ankle Joint" OR "Ankle" OR "Ankle joint" OR ankle)

**Central 🡪 87 hits**

**
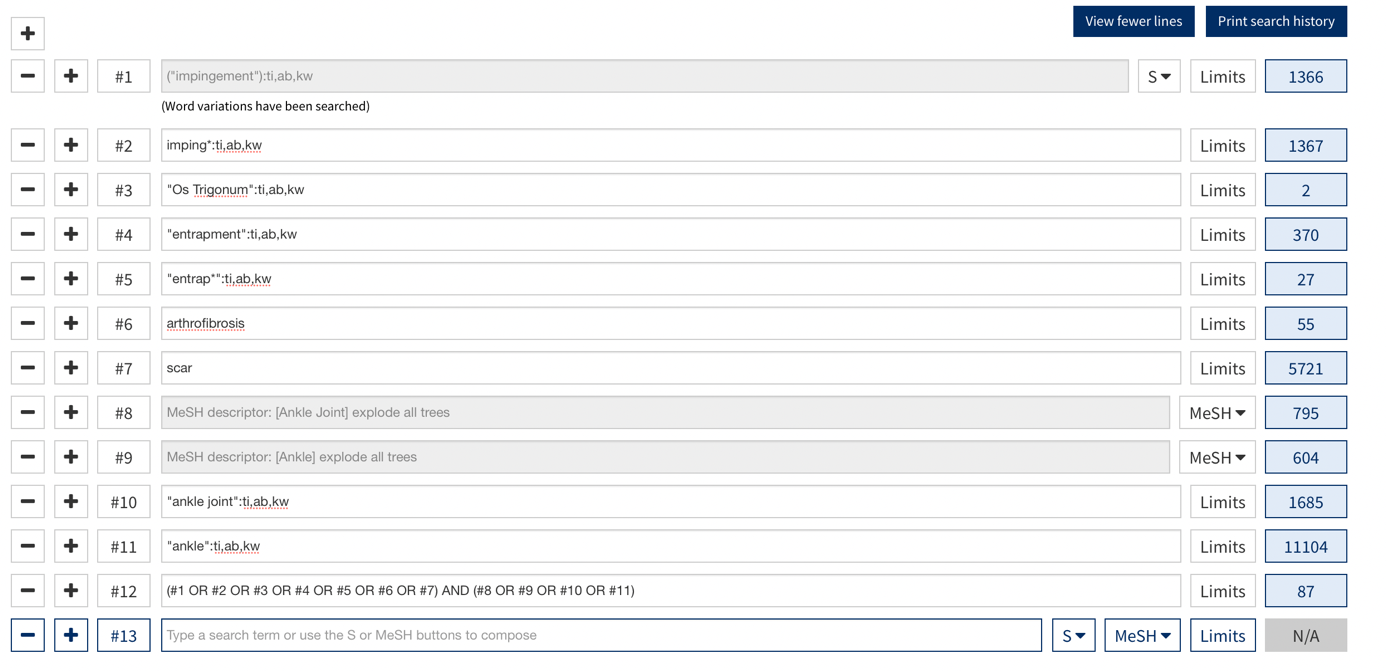
**

**Embase 🡪 1966 hits**

(impingement.mp

OR

(imping*.ab,kf,ti

OR

(os trigonum.ab,kf,ti

OR

(entrapment.ab,kf,ti

OR

(entrap*.ab,kf,ti

OR

(arthrofibrosis.ab,kf,ti

OR

(scar.ab,kf,ti)))))))

AND

(ankle joint.mp

OR

(ankle joint.ab,kf,ti

OR

(ankle.ab,kf,ti

OR

(ankle.ab,kf,ti))))
